# Supplementary material for: Molecular Characterization of Coxsackievirus A24v from Feces and Conjunctiva Reveals Epidemiological Links
Source: Microorganisms. 2021 Mar 5;9(3):531. doi: 10.3390/microorganisms9030531 (PMC7998715; doi:10.3390/microorganisms9030531)
Supplement: Supplementary file 1 [file microorganisms-09-00531-s001.pdf]

Supplementary Material

Molecular Characterization of Coxsackievirus A24v from Feces and Conjunctiva Reveals Epidemiological Links

Magilé C. Fonseca [1,*], Mario Pupo-Meriño [2], Luis A. García-González [3,4], Mayra Muné [1], Sonia Resik [1], Heléne Norder [5,6] and Luis Sarmiento [7,*]

[1] Virology Department, Center for Research Diagnosis, and Reference, Institute of Tropical Medicine “Pedro Kourí”, Havana 11400, Cuba; mayra@ipk.sld.cu (M.M.); sresik@ipk.sld.cu (S.R.)
[2] Departamento de Bioinformática, Universidad de las Ciencias Informáticas (UCI), Habana 19370, Cuba; mpupom@uci.cu
[3] Centro de Estudios de Matemática Computacional, Universidad de las Ciencias Informáticas (UCI), Habana 19370, Cuba; lgarciaag89@gmail.com
[4] Departamento de Ciencias de la Computación, Centro de Investigación Científica y de Educación Superior de Ensenada, 22860 Ensenada, Mexico
[5] Department of Infectious Diseases/Virology, Institute of Biomedicine, Sahlgrenska Academy, University of Gothenburg, 40530 Gothenburg, Sweden; helene.norder@gu.se
[6] Department of Clinical Microbiology, Region Västra Götaland, Sahlgrenska University Hospital,
41345 Gothenburg, Sweden
[7] Immunovirology Unit, Department of Clinical Sciences, Skåne University Hospital, Lund University, 22185 Malmo, Sweden
* Correspondence: magile@ipk.sld.cu (M.C.F.); luis.sarmiento-perez@med.lu.se (L.S.); Tel.: +537-255-35-50 (M.C.F.); +46-70-3067626 (L.S.)

Table S1. Number of the Cuban strains and specimens source, sequenced and included in the phylogenetic analysis for 3C (507nt) and VP1 (234nt) partial coding regions previously reported by Fonseca et.al [13]. Highlighted in grey the number of feces analyzed in this study.

Source: Fonseca et.el. 2019 [13]. CS: conjunctival swabs, F: feces, PS: pharyngeal swab,

AHC Cuban epidemics (years)	Number of sequenced strains/specimen sources of the strains	Number of sequenced obtained/ specimen sources of the strains		Number of sequenced selected for analysis after filtering duplex/specimen sources of the strains	
		3C	VP1	3C	VP1
1986	27 CS	27 CS	27 CS	3 CS	8 CS
1987	8 CS	6 CS	5 CS	1 CS	1 CS
1992	22 CS	22 CS	22 CS	9 CS	4 CS
1993	15 CS	15 CS	13 CS	7 CS	2 CS
1997	25 CS<br>16 F<br>1 PS<br>1 NS	25 CS<br>16 F<br>1 PS<br>1 NS	22 CS<br>14 F<br>1 PS<br>1 NS	6 CS<br>8 F	7 CS<br>2 F
2003	23 CS<br>16 F	23 CS<br>16 F	23 CS<br>16 F	9 CS<br>3 F	9 CS<br>1 F
2005	5 CS	5 CS	5 CS	1 CS	1CS
**Subtotal**	**159**	**157**	**149**	**47**	**35**
2008	5 CS<br>1 F	5 CS<br>1 F	*ND*	1 CS	*ND*
2009	9 CS<br>1 F	9 CS<br>1 F	*ND*	5 CS<br>1F	*ND*
**Total**	**175**	**173 (137 CS, 34 F, 1PS,1NS)**	**149 (117 CS, 30 F 1PS, 1 NS)**	**54 (42 CS,12 F)**	**35 (32 CS, 3F)**

NS: nasal swab, ND: non determined, the VP1 region was not sequenced

Table S2. Data set selected for phylogenetic analysis of the 3C coding region including world-wide and Cuban CVA24v sequences obtained from different specimens 1986-2009. The genotype is also shown. the sequences obtained from feces isolates of AHC patients are highlighted in yellow.

	GenBank accession number _strain name_country_year of isolation	Genotype
1	D90457_EH24_Singapore_1_1970	GI
2	D10296_Singapore_2_1970	GI
3	D10295_Singapore_3_1970	GI
4	D10297_Hong_Kong_1_China_1971	GI
5	D10298_Singapore_1_1975	GII
6	D10299_Singapore_2_1975	GII
7	D10300_Thailand_1_1975	GII
8	D10301_Singapore_1_1985	GIII
9	D10307_Okinawa_1_Japan_1985	GIII
10	D13270_L062_85_Taiwan_1985	GIII
11	D10267_Kanagawa_1_Japan_1986	GIII
12	D13273_V150_Taiwan_1986	GIII
13	D13272_V116_86_Taiwan_1986	GIII
14	FJ042688_Taiwan_1986	GIII
15	D10311_Okinawa_1_Japan_1986	GIII
16	D10303_Henan_1_China_1986	GIII
17	D10302_Shanghai_1_China_1986	GIII
18	D10315_Islamabad_Pakistan_1986	GIII
19	KC128616_CUBA_1986	GIII
20	KC128631_CUBA_1986	GIII
21	KC128638_CUBA_1986	GIII
22	KC128643_CUBA_1987	GIII
23	D10312_Ghana_1_1987	GIII
24	D10313_Ghana_2_1987	GIII
25	D10314_Ghana_3_1987	GIII
26	EF015037_Jamaica_1987	GIII
27	EF015038_Brazil_1987	GIII
28	GU983207_Brazil_1987	GIII
29	D10324_Kaohsiung_2_Taiwan_1988	GIII
30	D10322_Singapore_1_1988	GIII
31	D13278_590_Taiwan_1988	GIII
32	D13285_865_89_Taiwan_1989	GIII
33	D10277_Mobara_1_Japan_1989	GIII
34	AB008476_062_Taiwan_1990	GIII
35	KC205691_CUBA_1992	GIII
36	KC205693_CUBA_1992	GIII
37	KC205692_CUBA_1992	GIII
38	KC205695_CUBA_1992	GIII
39	KC205698_CUBA_1992	GIII
40	KC205702_CUBA_1992	GIII
41	KC205710_CUBA_1992	GIII
42	KC205711_CUBA_1992	GIII
43	KC205712_CUBA_1992	GIII
44	KC205713_CUBA_1993	GIII
45	KC205714_CUBA_1993	GIII

	GenBank accession number _strain name_country_year of isolation	Genotype
46	KC205715_CUBA_1993	GIII
47	KC205717_CUBA_1993	GIII
48	KC205720_CUBA_1993	GIII
49	KC205722_CUBA_1993	GIII
50	KC205726_CUBA_1993	GIII
51	AB008485_46_Thailand_1993	GIII
52	AB008484_26_Thailand_1993	GIII
53	EF015039_Dominican_Republic_1993	GIII
54	AB008481_072_Taiwan_1994	GIII
55	AB008480_066_Taiwan_1994	GIII
56	AB008479_063_Taiwan_1994	GIII
57	AB008477_042_Taiwan_1994	GIII
58	EF015040_USA_1998	GIV
59	KC286917_CUBA_1997	GIV
60	KC286918_CUBA_1997	GIV
61	KC286919_CUBA_1997	GIV
62	KC286924_CUBA_1997	GIV
63	KC286927_CUBA_1997	GIV
64	KC286937_CUBA_1997	GIV
65	KC286938_CUBA_1997	GIV
66	KC286940_CUBA_1997	GIV
67	KC286942_CUBA_1997	GIV
68	KC286945_CUBA_1997	GIV
69	KC286946_CUBA_1997	GIV
70	KC286947_CUBA_1997	GIV
71	KC286948_CUBA_1997	GIV
72	KC286949_CUBA_1997	GIV
73	DQ472129_Taiwan_2000	GIV
74	DQ472138_Taiwan_2000	GIV
75	DQ472136_Taiwan_2001	GIV
76	AB473409_Taiwan_2001	GIV
77	DQ472134_Taiwan_2002	GIV
78	DQ472137_Taiwan_2002	GIV
79	AY216777_South_Korea_2002	GIV
80	AY216782_South_Korea_2002	GIV
81	AY216789_South_Korea_2002	GIV
82	AY216790_South_Korea_2002	GIV
83	AY876913_Hangzhou13_02_China_2002	GIV
84	AY942640_Mumbai4_03_India_2003	GIV
85	AY942641_Pune6-03_India_2003	GIV
86	AY876182_French_Guiana_2003	GIV
87	AY876183_French_Guiana_2003	GIV
88	AY876186_Guadeloupe_2003	GIV
89	AY876187_Guadeloupe_2003	GIV
90	AY876191_Guadeloupe_2003	GIV
91	AY876192_Guadeloupe_2003	GIV
92	GU983208_Brazil_2003	GIV
93	GU983212_Brazil_2003	GIV
94	GU983221_Brazil_2003	GIV

	GenBank accession number _strain name_country_year of isolation	**Genotype**
95	GU983230_Brazil_2004	GIV
96	GU983232_Brazil_2004	GIV
97	KC286994_CUBA_2003	GIV
98	KC286998_CUBA_2003	GIV
99	KC287000_CUBA_2003	GIV
100	KC287005_CUBA_2003	GIV
101	KC287010_CUBA_2003	GIV
102	KC287011_CUBA_2003	GIV
103	KC287012_CUBA_2003	GIV
104	KC287013_CUBA_2003	GIV
105	KC287014_CUBA_2003	GIV
106	KC287016_CUBA_2003	GIV
107	KC287022_CUBA_2003	GIV
108	KC287025_CUBA_2003	GIV
109	KC287037_CUBA_2005	GIV
110	DQ370152_0382_04_DR_Congo_2004	GIV
111	DQ370147_0370_04_DR_Congo_2004	GIV
112	DQ370146_0365_04_DR_Congo_2004	GIV
113	DQ370162_01531_04_Morocco_2004	GIV
114	DQ370160_01529_04_Morocco_2004	GIV
115	DQ370159_01525-04_Morocco_2004	GIV
116	DQ443002_Singapore_2005	GIV
117	DQ443001_Singapore_2005	GIV
118	GU983236_Brazil_2005	GIV
119	GU983238_Brazil_2005	GIV
120	FJ200506_Taiwan_2006	GIV
121	FJ232037_Taiwan_2006	GIV
122	FJ200514_Taiwan_2007	GIV
123	FJ042700_Taiwan_2007	GIV
124	FJ042701_Taiwan_2007	GIV
125	GU477563_India_2007	GIV
126	GU477564_India_2007	GIV
127	GU477568_India_2007	GIV
128	HM156623_CUBA_2008	GIV
129	HM156613_CUBA_2009	GIV
130	HM156616_CUBA_2009	GIV
131	HM156617_CUBA_2009	GIV
132	HM156618_CUBA_2009	GIV
133	HM156621_CUBA_2009	GIV
134	HM156622_CUBA_2009	GIV
135	GU983241_Brazil_2009	GIV
136	GU983242_Brazil_2009	GIV
137	GU983243_Brazil_2009	GIV

Table S3. Data set selected for phylogenetic analysis of the VP1 coding region including world-wide and Cuban CVA24v sequences obtained from different specimens 1986-2009. The Cuban sequences obtained from feces isolates of AHC patients (n=3) are highlighted in green; sequences obtained from Synanthropic nonhuman primates feces isolates (n=3) are highlighted in yellow , the sequences obtained from the AFP feces isolates (n=27) are highlighted in orange, and the sequences obtained from Philippines rivers samples (n=5) are highlighted in grey.

	**GenBank accession number _strain name_country_year of isolation**	**Genotype**

1	D90457_EH24_70_Singapore_1970	GI
2	AB473429_L001_Taiwan_1985	GIII
3	AB473430_V150_Taiwan_1986	GIII
4	KC184859_CUBA_1986	GIII
5	KC184869_CUBA_1986	GIII
6	KC184872_CUBA_1986	GIII
7	KC184873_CUBA_1986	GIII
8	KC184875_CUBA_1986	GIII
9	KC184877_CUBA_1986	GIII
10	KC184879_CUBA_1986	GIII
11	KC184881_CUBA_1986	GIII
12	KC184886_CUBA_1987	GIII
13	EF015038_Brazil_1987	GIII
14	GU983190_PA_1_Brazil_1987	GIII
15	EF015037_Jamaica_1987	GIII
16	AB473432_865_Taiwan_1989	GIII
17	AB473431_804_Taiwan_1989	GIII
18	EF015039_Dominican_Republic_1993	GIII
19	KC205656_CUBA_1991	GIII
20	KC205664_CUBA_1992	GIII
21	KC205675_CUBA_1992	GIII
22	KC205676_CUBA_1992	GIII
23	KC205682_CUBA_1993	GIII
24	KC205690_CUBA_1993	GIII
25	95_PHL_1996_Philippines_1996	GIV
26	107_PHL_1996_Philippines_1996	GIV
27	174_PHL_1996_Philippines_1996	GIV
28	111_PHL_1997_Philippines_1997	GIV
29	KC286959_CUBA_1997	GIV
30	KC286960_CUBA_1997	GIV
31	KC286963_CUBA_1997	GIV
32	KC286966_CUBA_1997	GIV
33	KC286972_CUBA_1997	GIV
34	KC286977_CUBA_1997	GIV
35	KC286981_CUBA_1997	GIV
36	KC286983_CUBA_1997	GIV
37	KC286988_CUBA_1997	GIV
38	EF015040_Texas_USA_1998	GIV
39	AY208105_98_30257_38_99_France_1998	GIV
40	AB473433_Taiwan_2000	GIV
41	14_PHL_2000_Philippines_2000	GIV
42	15_PHL_2000_Philippines_2000	GIV
43	28_PHL_2000_Philippines_2000	GIV
	**GenBank accession number _strain name_country_year of isolation**	**Genotype**
44	30_PHL_2000_Philippines_2000	GIV
45	31_PHL_2000_Philippines_2000	GIV
46	159_PHL_2000_Philippines_2000	GIV
47	AB473434_Taiwan_2001	GIV
48	AB473435_Taiwan_2001	GIV
49	63_PHL_2002_Philippines_2002	GIV
50	172_PHL_2002_Philippines_2002	GIV

51	DQ434857_South_Korea_2002	GIV
52	DQ434858_South_Korea_2002	GIV
53	DQ434861_South_Korea_2003	GIV
54	DQ434866_South_Korea_2003	GIV
55	GQ329725_China_2003	GIV
56	AY876169_French_Guiana_2003	GIV
57	AY876174_Guadeloupe_2003	GIV
58	AY876175_Guadeloupe_2003	GIV
59	AY876179_Guadeloupe_2003	GIV
60	GU983184_Brazil_2003	GIV
61	GU983186_Brazil_2003	GIV
62	GU983187_Brazil_2003	GIV
63	GU983193_Brazil_2004	GIV
64	GU983196_Brazil_2004	GIV
65	KC287038_CUBA_2003	GIV
66	KC287040_CUBA_2003	GIV
67	KC287048_CUBA_2003	GIV
68	KC287049_CUBA_2003	GIV
69	KC287053_CUBA_2003	GIV
70	KC287054_CUBA_2003	GIV
71	KC287055_CUBA_2003	GIV
72	KC287056_CUBA_2003	GIV
73	KC287058_CUBA_2003	GIV
74	KC287064_CUBA_2003	GIV
75	KC287080_CUBA_2005	GIV
76	AB365074_Pakistan_2004	GIV
77	AB365075_Pakistan_2004	GIV
78	AB365076_Pakistan_2004	GIV
79	EU162074_Spain_2004	GIV
80	EU162077_Spain_2004	GIV
81	GU983199_Brazil_2005	GIV
82	GU983201_Brazil_2005	GIV
83	GU983203_Brazil_2005	GIV
84	DQ443001_Singapore_2005	GIV
85	DQ443002_Singapore_2005	GIV
86	DQ901736_Singapore_2005	GIV
87	FJ232034_Taiwan_2006	GIV
88	AB473436_Taiwan_2006	GIV
89	EF176672_South_Korea_2006	GIV
90	EF176676_South_Korea_2006	GIV
91	FJ868371_Australia_2006	GIV
92	GU477582_India_2007	GIV
93	GU477573_India_2007	GIV
	GenBank accession number _strain name_country_year of isolation	Genotype
94	GU477579_India_2007	GIV
95	GU477580_India_2007	GIV
96	GQ229398_China_2007	GIV
97	EU391662_China_2007	GIV
98	GQ429279_China_2007	GIV
99	AB473439_Taiwan_2007	GIV
100	AB473440_Taiwan_2007	GIV

101	AB473441_Taiwan_2007	GIV
102	JX538080_14662_Bangladesh_2007	GIV
103	JX538079_14661_Bangladesh_2007	GIV
104	GQ429280_China_2008	GIV
105	GQ429287_China_2008	GIV
106	GQ429288_China_2008	GIV
107	JQ744321_30_Ma_223_PHL_Philippines_2008	GIV
108	JQ744322_33_LP_172_PHL_Philippines_2008	GIV
109	JQ744323_30_Ma_221_PHL_Philippines_2008	GIV
110	JQ744324_20_Pa_235_PHL_Philippines_2008	GIV
111	JQ744317_38_Me_271_PHL_Philippines_2008	GIV
112	128_PHL_2008_Philippines_2008	GIV
113	JX538127_709011_Bangladesh_2008	GIV
114	JX538128_709012_Bangladesh_2008	GIV
115	JX538132_709041_Bangladesh_2008	GIV
116	JX538205_14793_Bangladesh_2008	GIV
117	JX538207_14795_Bangladesh_2008	GIV
118	JX538211_14806_Bangladesh_2008	GIV
119	JX538213_14808_Bangladesh_2008	GIV
120	JX538214_14809_Bangladesh_2008	GIV
121	JX538216_14815_Bangladesh_2008	GIV
122	JX538220_14820_Bangladesh_2008	GIV
123	JX417879_24v_g08_005_Gabon_2008	GIV
124	KF667358_INDNIV1034661LV463_India_2009	GIV
125	KF667359_INDNIV1036731LV476_India_2009	GIV
126	KF667360_INDNIV1044161LV530_India_2009	GIV
127	KF667361_INDNIV1040633LV639_India_2009	GIV
128	GU983204_Brazil_2009	GIV
129	GU983205_Brazil_2009	GIV
130	GU983206_Brazil_2009	GIV

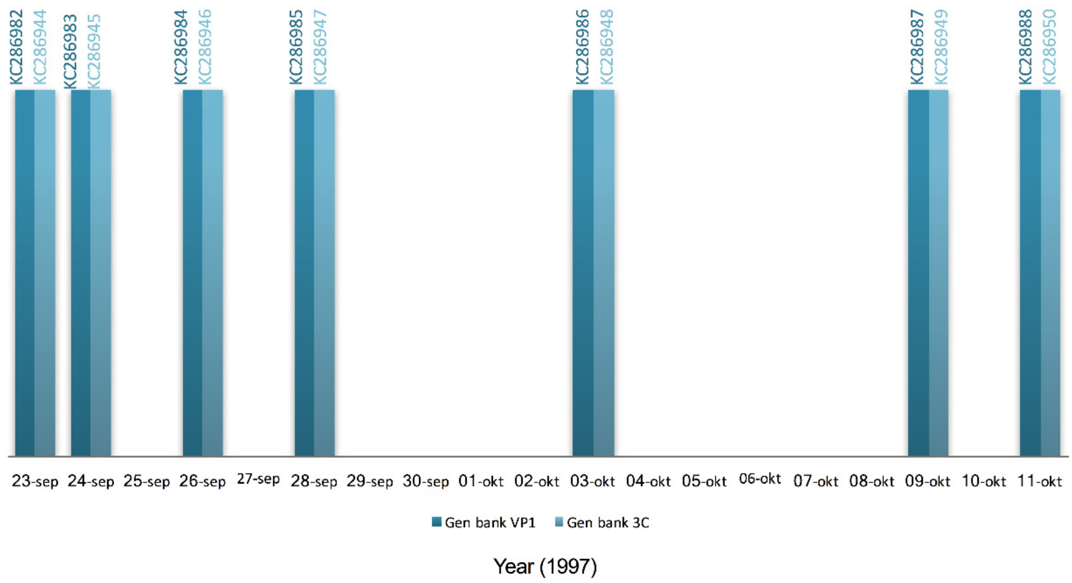

Figure S1. Dates for each VP1 and 3C sequence of CVA24v strains isolated from the serial feces samples from one patient.
